# A Uterine Leiomyoma With a Novel BRAF::ABCB1 t(7; 7)(q34; q21.12) Fusion: A Case Report

**DOI:** 10.7759/cureus.106965

**Published:** 2026-04-13

**Authors:** Cao Jin, Tahyna Hernandez, Sudarshana Roychoudhury

**Affiliations:** 1 Pathology, Northwell Health, New Hyde Park, USA; 2 Pathology and Laboratory Medicine, Northwell Health, New Hyde Park, USA

**Keywords:** benign tumor, braf gene, leiomyoma variant, med12, smooth muscle tumor

## Abstract

Uterine leiomyomas are the most common benign neoplasms in women, typically driven by mutations in *MED12*. We report the case of a 68-year-old woman with a history of uterine fibroids who underwent a total laparoscopic hysterectomy. Microscopically, the tumor was a benign-appearing spindle cell neoplasm with focal edema, hyaline plaques, and hemorrhage, but lacking high-grade atypia, tumor cell necrosis, or significant mitotic activity. Immunohistochemistry was consistent with a smooth muscle origin (positive for desmin and smooth muscle actin (SMA), negative for CD10). Due to its unusual features on imaging, next-generation RNA sequencing was performed, which identified a novel *BRAF*::*ABCB1* fusion secondary to t(7;7)(q34;q21.12). This case describes a new, potentially targetable molecular alteration in a uterine leiomyoma, distinct from the common *MED12*-mutated pathway, highlighting the underlying molecular heterogeneity of these common tumors.

## Introduction

Uterine leiomyomas, or fibroids, are benign smooth muscle tumors of the uterus and represent the most common neoplasms in women of reproductive age. While benign, they can cause significant morbidity, including abnormal uterine bleeding, pelvic pain, and infertility, impacting millions of women globally [1,2].

Approximately 40-50% of uterine leiomyomas (fibroids) exhibit non-random, tumor-specific chromosomal abnormalities [1]. The molecular pathogenesis of leiomyomas is heterogeneous, but a majority (over 70%) are driven by somatic mutations in the *MED12* gene, particularly in exon 2, which encodes a subunit of the Mediator complex crucial for gene transcription [3]. Other less frequent genetic drivers have been well-characterized, including rearrangements of *HMGA2*, biallelic inactivation of *FH*, or mutations in* COL4A5/COL4A6*, defining distinct molecular subtypes [4]. Each of these distinct genetic alterations defines a specific molecular subtype of uterine leiomyoma, suggesting varied pathways of tumor initiation and progression.

Against this backdrop of established genetic drivers, this particular report describes a singular case of a uterine leiomyoma. Intriguingly, this tumor exhibited conventional morphology but harbored a novel and previously unreported genetic anomaly: a *BRAF::ABCB1 *gene fusion. This finding further expands our understanding of the genetic landscape and molecular heterogeneity of uterine leiomyomas.

## Case presentation

Clinical history

A 68-year-old female patient with a past medical history of hypertension, hyperlipidemia, type 2 diabetes mellitus, and seasonal allergies presented for an annual gynecologic visit. She had a known history of uterine fibroids for 17 years, which had recently increased in size, but she was asymptomatic at the time of presentation. She had not been on any hormone therapy. A recent magnetic resonance imaging (MRI) study was performed to investigate her symptoms of pelvic pain and abnormal uterine bleeding, which prompted evaluation for surgical management. The patient subsequently underwent a total laparoscopic hysterectomy with bilateral salpingo-oophorectomy.

Radiologic findings

**Figure 1:**
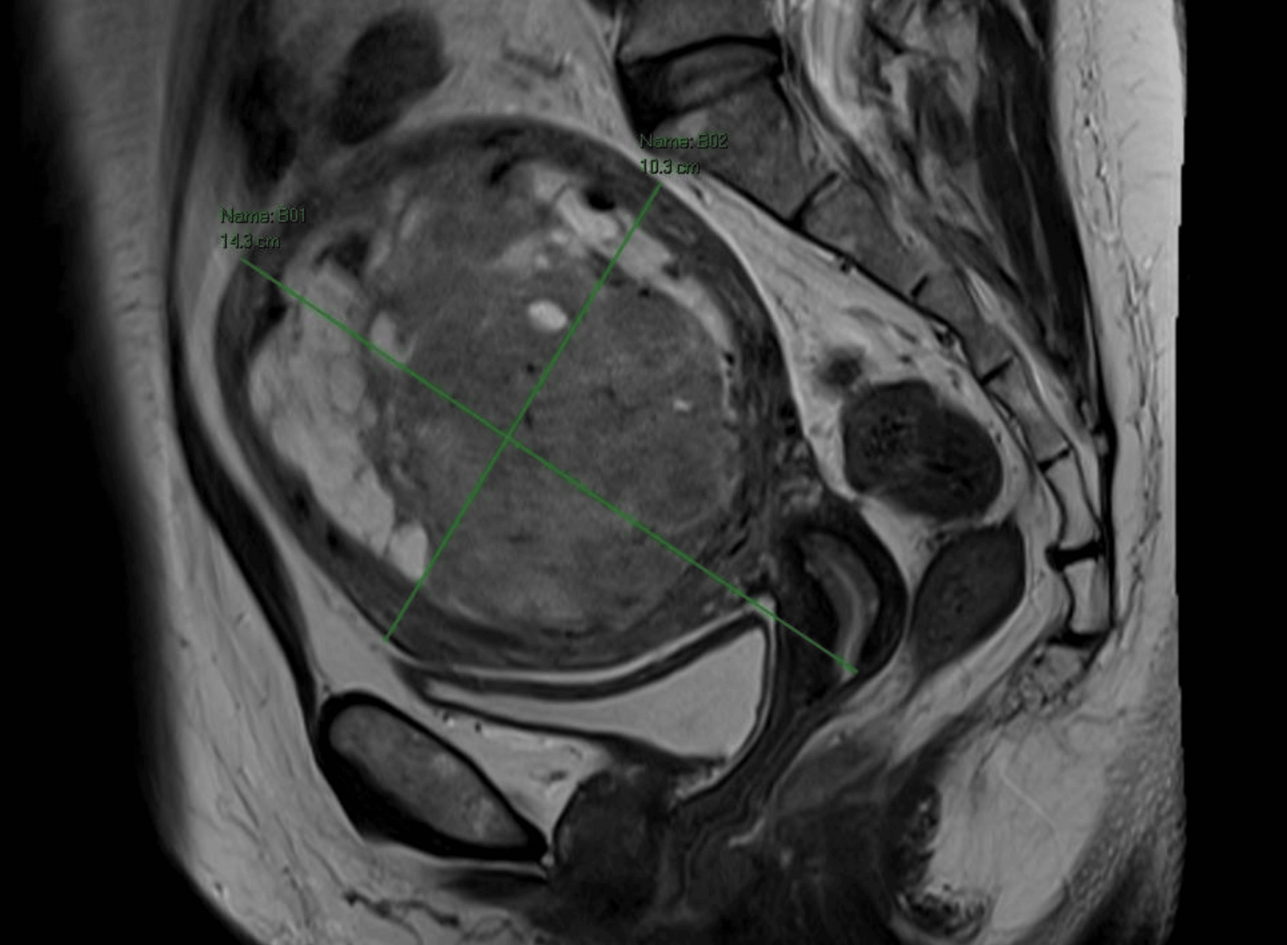
MRI pelvis T2 sagittal view showed enlarged heterogenous fibroid prominent nonenhancing cystic spaces

The MRI revealed an enlarged fibroid uterus measuring 14.3 x 10.3 x 10.1 cm. A solitary posterior body intramural fibroid was noted, measuring 10.4 x 9.0 x 9.1 cm. The fibroid was T2 heterogeneous with prominent nonenhancing cystic spaces, features compatible with cystic degeneration (Figure 1).

Pathologic findings

The hysterectomy specimen, including the uterus and bilateral adnexa, weighed 426.3 g. Gross examination revealed multiple disrupted, tan-white to yellow, whorled tissue fragments, the largest measuring 9.0 x 4.0 x 3.5 cm. Sectioning of the dominant mass showed a diffusely tan-yellow to gray, whorled cut surface. Focal dark red to brown hemorrhagic areas were present, constituting approximately 10% of the cut surface. The endometrium was grossly unremarkable.

**Figure 2:**
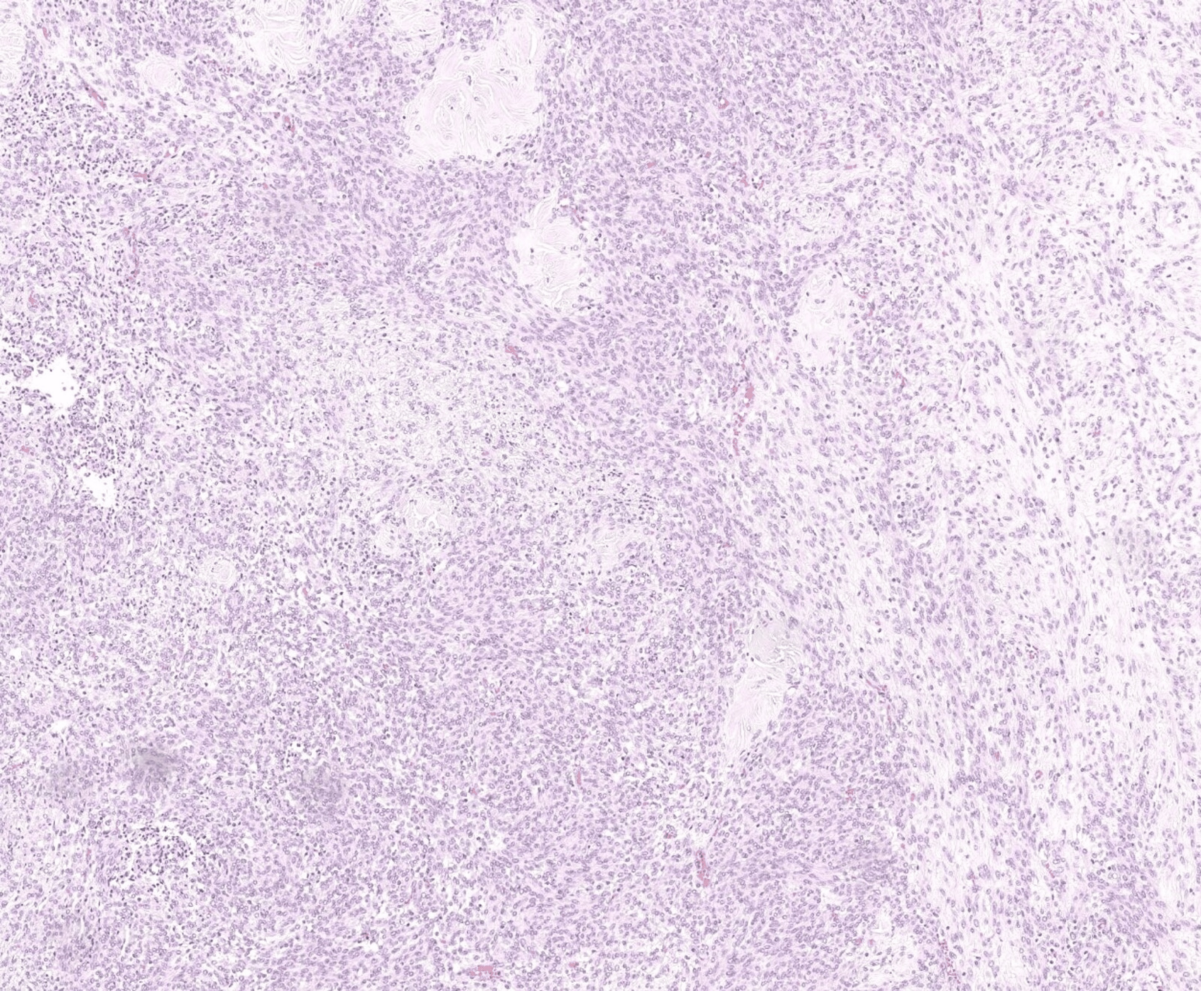
At 40x, a spindle and epithelioid cell neoplasm is seen with focal edema and extensive hyaline plaques

**Figure 3:**
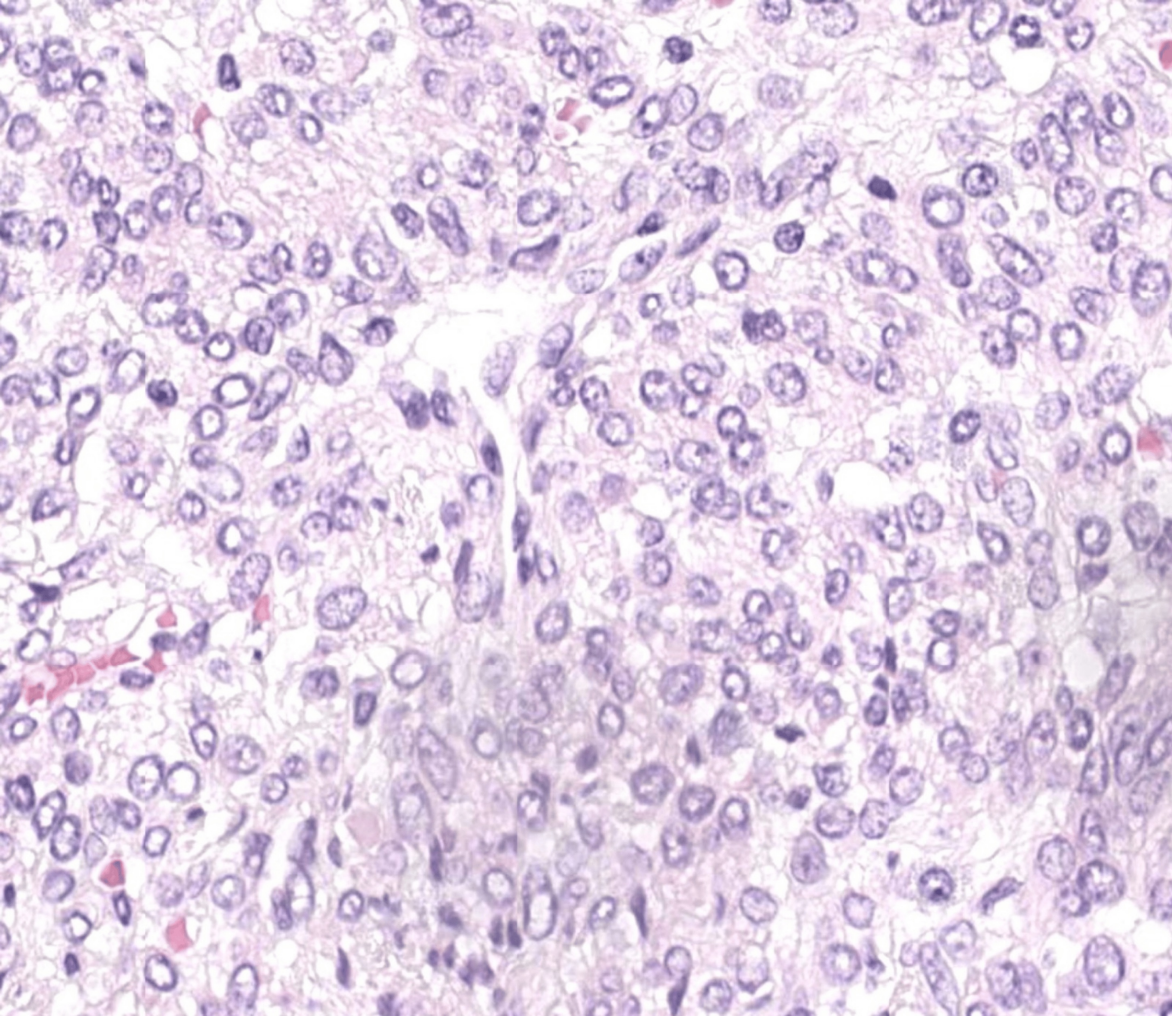
At 400x, high power, the tumor does not show any moderate or severe atypia, nor mitosis

Microscopic examination showed a spindle and epithelioid cell neoplasm arranged in fascicles. The tumor exhibited focal edema and extensive hyaline plaques. (Figures 2, 3) Focal hemorrhage and calcifications were also identified. Crucially, features of malignancy such as high-grade nuclear atypia, tumor cell necrosis, and increased mitotic activity were not identified.

**Figure 4:**
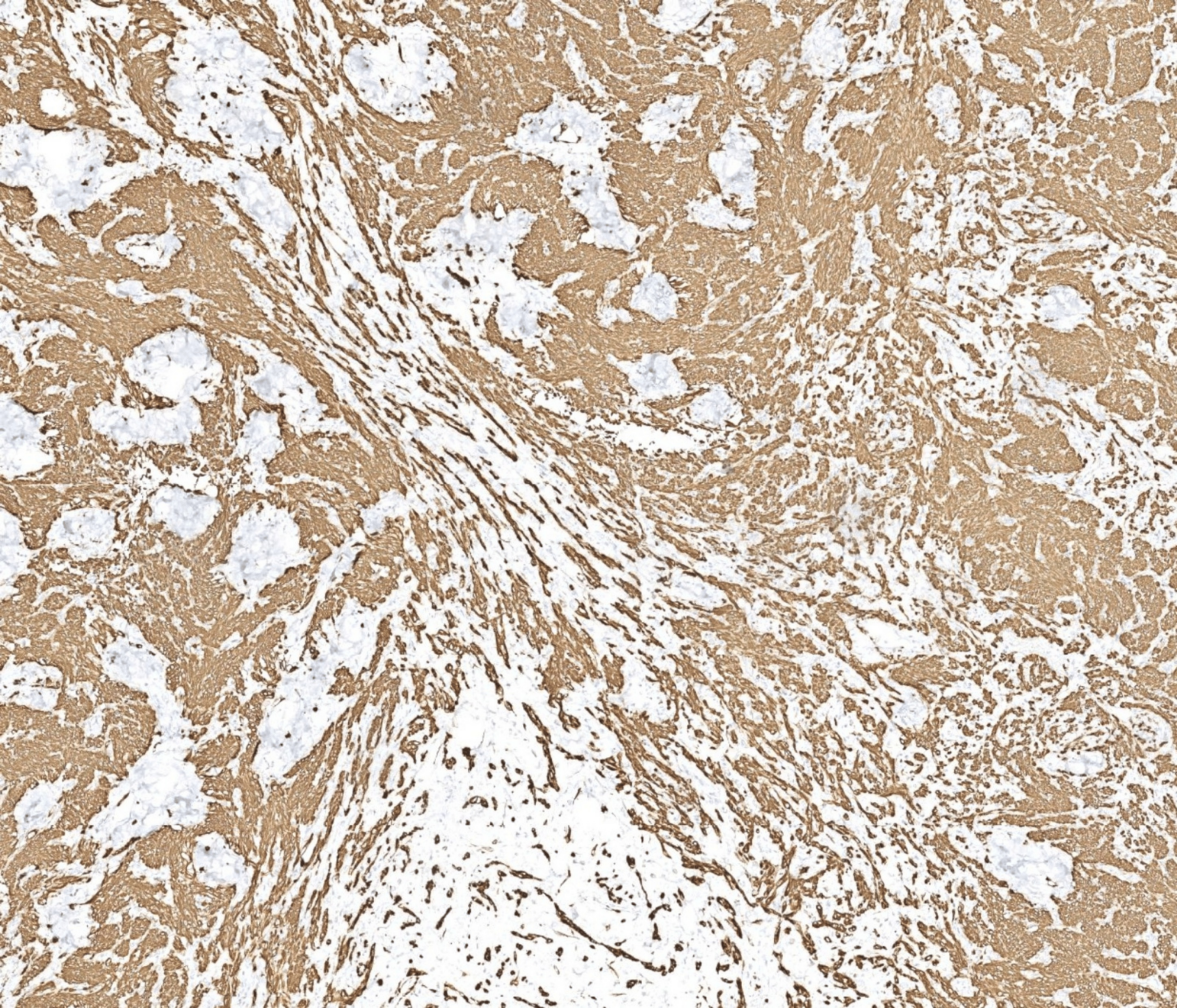
At 40x, immunostain for desmin is strongly positive in the tumor.

**Figure 5:**
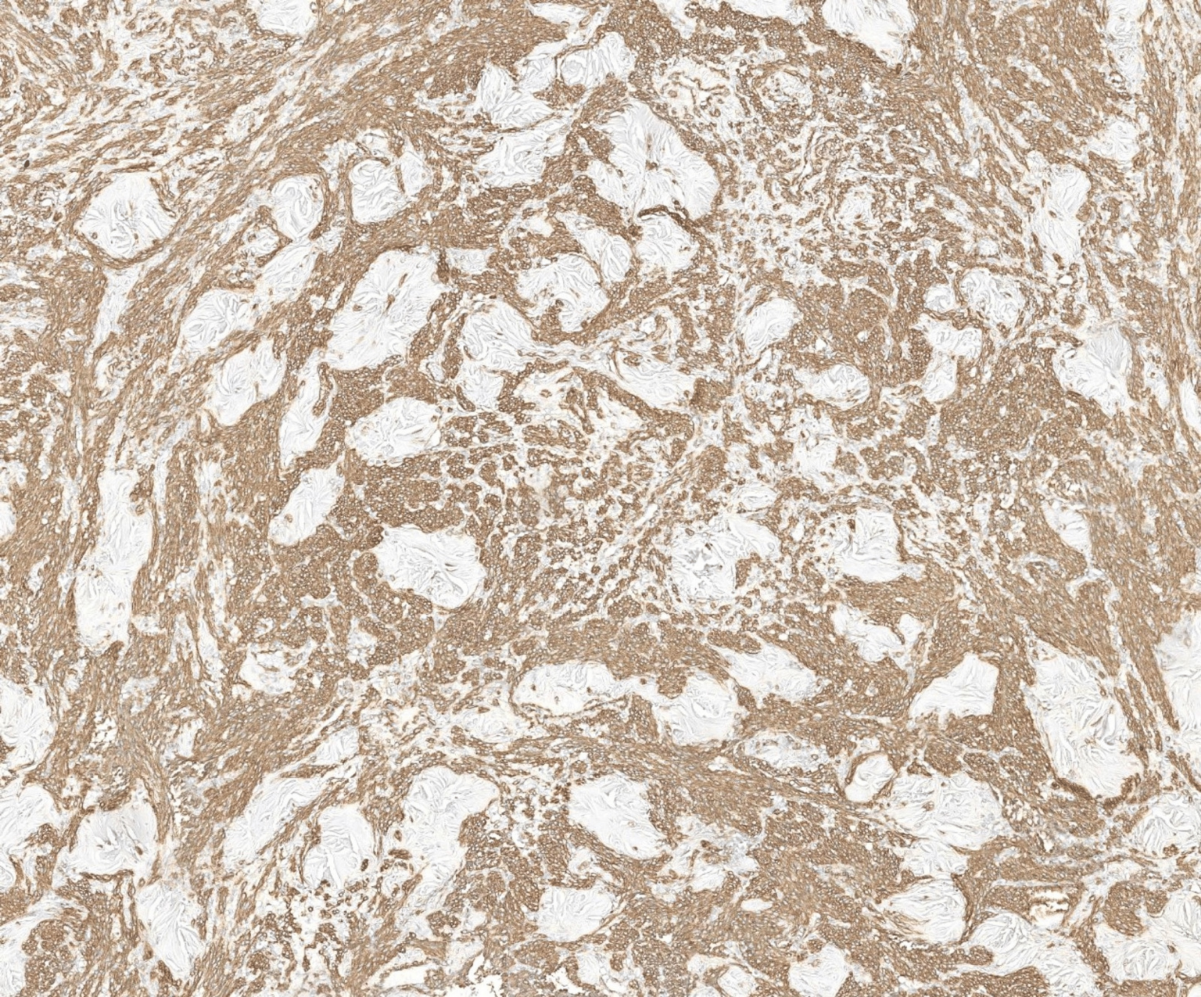
At 40x, immunostain for SMA is strongly positive in the tumor SMA: smooth muscle actin

Immunohistochemical stains were performed, showing the tumor cells were strongly and diffusely positive for desmin and smooth muscle actin (SMA), and predominantly negative for CD10. This immunoprofile supported a diagnosis of a smooth muscle neoplasm (Figures 4, 5)

Molecular findings

Given the large size and degenerative features, a sample of the tumor was submitted for a targeted RNA-based next-generation sequencing (NGS) panel designed to detect several hundred known gene fusions associated with sarcomas and other mesenchymal tumors, as well as novel fusions involving targeted oncogenes and tumor suppressors. The analysis revealed a novel *BRAF::ABCB1* fusion secondary to t(7;7)(q34;q21.12). This translocation was inferred from the RNA sequencing data, as conventional karyotyping was not performed. The initial diagnosis of "uterine leiomyoma" was revised to "uterine leiomyoma with a *BRAF::ABCB1* fusion" to incorporate the molecular finding.

## Discussion

This was a case of a morphologically benign uterine leiomyoma harboring a novel *BRAF::ABCB1* gene fusion. This finding adds a new alteration to the known spectrum of genetic alterations in uterine smooth muscle tumors and adds to the growing molecular characterization of *MED12*-wildtype leiomyomas [3]. While *BRAF *V600E point mutations are known oncogenic drivers in diverse malignancies [5], gene fusions involving the BRAF kinase domain are less common but recognized oncogenic events in tumors such as papillary thyroid carcinoma (*AKAP9::BRAF*) and various sarcomas [6,7]. Our finding of a *BRAF* fusion in a uterine leiomyoma suggests that mitogen-activated protein kinase (MAPK) pathway activation may be a rare but alternative path to tumorigenesis in these common benign neoplasms.

The proposed primary oncogenic mechanism of *BRAF* fusions is the constitutive, ligand-independent activation of the MAPK signaling pathway. This is typically achieved when the N-terminal autoinhibitory domain of *BRAF* is replaced by a fusion partner that provides a dimerization or oligomerization domain, forcing the BRAF kinase into an active conformation [7]. In this case, the intra-chromosomal rearrangement t(7;7)(q34;q21.12) juxtaposes the BRAF kinase domain with a portion of the *ABCB1* gene. The *ABCB1* gene, also known as MDR1, encodes the P-glycoprotein efflux pump, a transmembrane protein whose overexpression is a classic mechanism of multidrug resistance in cancer [8]. It is most likely that the *ABCB1* component of the fusion serves as a structural partner, providing the necessary dimerization motif to drive constitutive *BRAF *activation and subsequent cellular proliferation.

Interestingly, despite being potentially driven by a potent oncogenic fusion, the tumor remained morphologically benign, lacking atypia, necrosis, or significant mitotic activity. This highlights the critical role of cellular context in determining tumor phenotype.

The identification of a targetable alteration in a leiomyoma has potential future clinical implications. Currently, uterine leiomyomas are not routinely treated with personalized therapy based on specific genetic mutations (such as *MED12*), despite advancements in identifying these drivers. Treatment remains largely focused on symptom management, including hormone therapy, uterine artery embolization (UAE), and surgery, based on tumor size and location, rather than genetic profiling [1,2].

The discovery of a *BRAF* fusion introduces the possibility of a completely different therapeutic approach. The success of BRAF inhibitors in treating* BRAF*-mutant melanoma has established the paradigm of targeting this pathway [9]. While not relevant for this surgically cured patient, the identification of such alterations is notable. For patients with symptomatic, *BRAF*-fusion-positive leiomyomas who are poor surgical candidates or desire fertility-sparing alternatives, treatment with a *BRAF* inhibitor could one day represent a novel, non-hormonal, targeted therapy, assuming the fusion is proven to be a functional driver. This aligns with the broader movement toward personalized medicine for uterine fibroids based on their specific molecular drivers [10]. Such implications have also been observed in prior genetically profiled leiomyomas. For example, *MED12*-mutant fibroids show reduced response to gonadotropin-releasing hormone (GnRH) agonists (lower volume reduction rate) and are less sensitive to progesterone receptor modulators compared to other types [11]. *HMGA2* overexpression leiomyoma iare highly dependent on the insulin-like growth factor (IGF) signaling pathway, making IGF inhibitors potential candidates [12]. *FH* deficiency leiomyoma may be linked to hereditary leiomyomatosis and renal cell cancer (HLRCC) syndrome, and identification of such leiomyoma could offer early screening options to patients [13]. 

This case report is limited by its nature as a single observation. Since this is the first case report about *BRAF* gene fusion in leiomyoma, the prognostic value of this fusion is unknown. Also, the DNA sequencing for *MED12* mutations was not performed, and therefore, the *MED12* mutation status of this tumor remains unknown. The immediate next steps should be to screen larger cohorts of uterine leiomyomas, particularly those that are large, unusual, or lack *MED12 *mutations, to determine the prevalence of *BRAF* fusions. Functional studies are also crucial to confirm that the *BRAF::ABCB1* fusion protein does indeed activate the MAPK pathway and to test its sensitivity to *BRAF* and/or MEK inhibitors in vitro. Correlating this genotype with long-term clinical data will be essential to understand if this subtype has a distinct natural history regarding growth rate or recurrence.

## Conclusions

The *BRAF::ABCB1* fusion represents a novel molecular alteration in uterine leiomyoma. It is for the first time that such fusion has been reported in leiomyoma. This finding expands our understanding of leiomyoma, which is not one single entity, but it is indeed a tumor comprised of various underlying genetic features, which may help explain different clinical behaviors of leiomyoma. In addition, identification of the *BRAF::ABCB1* fusion may offer a potentially targetable pathway, opening an exciting new avenue for the development of precision therapies for leiomyoma beyond conventional therapy. 
